# In Vitro Assessment of Optical Properties of Blood by Applying the Extended Huygens-Fresnel Principle to Time-Domain Optical Coherence Tomography Signal at 1300 nm

**DOI:** 10.1155/2008/591618

**Published:** 2008-07-07

**Authors:** Dan P. Popescu, Michael G. Sowa

**Affiliations:** National Research Council of Canada, Institute for Biodiagnostics, 435 Ellice Avenue, Winnipeg, MB, Canada R3B 1Y6

## Abstract

A direct method for the measurement of the optical attenuation coefficient and the scattering anisotropy parameter based on applying the extended Huygens-Fresnel principle to optical coherence tomography images of blood is demonstrated. The images are acquired with a low-power probing beam at the wavelength of 1300 nm. Values of 12.15 mm^−1^ and 0.95 are found for the total attenuation coefficient and the scattering anisotropy factor, respectively. Also, as a preliminary step, the optical refraction index is determined with a precision of two decimal numbers directly from optical coherence images. The total attenuation coefficient and the scattering anisotropy factor are determined with precisions within experimental error margins of 5% and 2%, respectively. Readable OCT signal is obtained for a maximum propagation of light into blood of 0.25 mm. At the maximum probed depth, the measured signal is almost 10^3^ smaller than its initial intensity when entering the sample.

## 1. INTRODUCTION

Precise measurements of the optical properties of biological tissues and fluids are important for a large class of medical diagnostics as well as therapeutic and surgical approaches that employ light. Often blood is present and therefore its optical properties need to be considered in the design or deployment of these optical methods [1–4]. Given its complexity as a propagating environment for light, a wide array of experimental investigations into the optical properties of blood has been carried out. There are methods that directly estimate the light transmission and scattering properties of blood but they can be applied only to thin blood samples, require complicated optical setups, and are suitable only for *ex-vivo* situations [5, 6]. A number of studies have been published about blood properties based on widely used indirect methods of modeling light propagation in turbid environments: wave-scattering theory [7, 8], photon-diffusion theory [1, 9], and inverse Monte-Carlo simulations [10, 11]. Although extensive and rigorous in the mathematical sense, the results depend on a precise model of the tissue structures as well as the optical properties associated with these structures. Often simplified tissue model geometries are employed in these cases to render analytical or computationally tractable solutions and, when coupled with imprecise knowledge of the various optical parameters, the accuracy of these indirect methods can be questioned. In this paper, we demonstrate a simple and efficient method to directly determine a number of clinically relevant optical parameters such as the index of refraction, the attenuation coefficient, and the scattering anisotropy factor of blood. These parameters are measured at 1300 nm, a promising wavelength to be used in clinical devices for probing highly scattering biological samples because it is scattered less than light at shorter wavelengths. The estimations of the attenuation coefficient and of the anisotropy factor are based on a numerical algorithm fit on the experimentally acquired optical coherence tomography (OCT) heterodyne efficiency curve. The connection between these parameters characterizing blood and the dependence on the propagation distance within blood of the detected OCT signal is provided by the extended Huyghens-Fresnel principle [12]. Previously, this optical method has been used successfully in determining the scattering properties of various biological samples as well as phantom probes of blood [13–16]. The OCT measurements presented here are acquired in fresh blood that flows freely within a system of flow cells. The results obtained in this configuration could be relevant in designing and using intravascular OCT catheters. Also, the viability of the extended Huygens-Fresnel principle is verified for OCT probing beams at low powers. Checking the viability of this principle at low intensities is necessary because in clinical settings the investigating beam could be strongly quenched while passing through other tissues before reaching the region of interest.

## 2. EXPERIMENT

OCT images are recorded using radiation emitted from a super-luminescent SLD-571-HP diode with the central wavelength at 1300 nm and a coherence length of 14 *μ*m (full width at half maximum) measured in air. Due to its lower absorption and scattering losses in tissues when compared to visible and shorter near-infrared wavelengths, the spectral region around 1300 nm has the potential to become important for a number of biophotonics applications [17–19]. In both arms of the interferometer, sample and reference, light is guided through single-mode fibers terminated with collimating lenses. In the reference arm, a rapid scanning optical delay line with a constant velocity of 600 mm/s modulates the optical field. A weakly focusing lens with a 48-mm focal distance and a focal depth of 1.6 mm is located near the exit of the sample fiber for the purpose of focusing the collimated light onto the sample. The estimated numerical aperture of the sample arm optical configuration is 0.025. Such a small value of the numerical aperture together with the proper positioning of the probed region within the depth of focus provides a beam that could be approximated as collimated along the axial section of the scanned volume. This configuration ensures that the beam divergence does not influence the intensity of the OCT signal along the depth of interest. The fiber-lens assembly is mounted on a computer-controlled horizontal translation stage that can scan the focused spot of the illuminating beam along the surface of interest with a maximum spatial resolution of 1 *μ*m. The power of the beam exiting the sample arm of the interferometer is 2 mW.

Fresh porcine blood is acquired from an abattoir immediately after sacrifice. Using a Masterflex pump, blood is continuously circulated in a closed-loop circuit through two consecutive 48/Q/2 Starna flow cells at a constant flow rate of 1 mL/min. One cell has the thickness of its flowing channel of 2 mm while the other flow channel in the other cell is 0.2 mm wide. Blood is flowing normal to the propagation direction of light exiting the focusing lens that terminates the sample arm of the OCT system. The measured haematocrit, that is, the ratio of the volume of red blood cells to the entire volume of blood, stayed constant at 44% for the duration of the experiment. In order to avoid coagulation, 2 mL/L of heparin was added to the blood immediately after acquisition. Heparin was added at a rate of 1 mL/L at 1-hour intervals for the remainder of the protocol. The blood was kept at room temperature for the duration of the experiment.

## 3. RESULTS AND DISCUSSION

**Figure 1:**
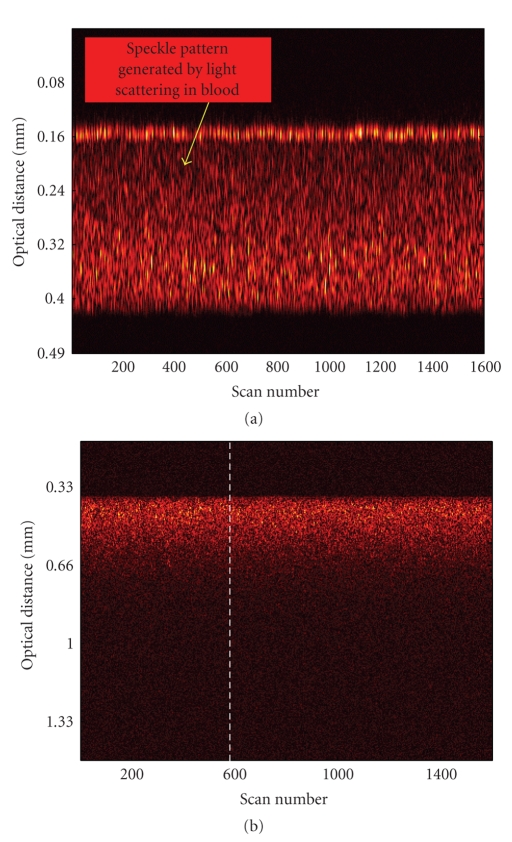
(a) OCT image of blood flowing through the 0.2-mm cell. Both blood/glass interfaces are visible. (b) OCT image of blood flowing through the 2-mm cell. The interrupted white line marks the 600th A-scan. Both images are composed of 1600 A-scans.

The OCT image presented in Figure 1(a) is obtained by acquiring a set of 1600 consecutive depth scans (also known as line scans or A-scans). This image shows the detected signal backscattered by blood cells that are flowing through the 0.2-mm cell during its acquisition time. To eliminate the random thermal, mechanical, and electronic noise, each depth scan is averaged 10 times. The acquisition time required for such an image is fifteen minutes. The image presents itself as a speckled pattern that is generated by the portion of the light flux that reaches the OCT detection system after it is scattered at the interfaces of blood cells with the surrounding plasma. This image shows the entire cross-section of the flow channel crossing through the 0.2-mm cell. In this case, both blood and glass interfaces are distinguishable and the optical distance between them can be assessed directly from the OCT image. Knowing the width of the flow channel, the value of the optical refraction index of blood is readily obtained by dividing the optical distance between the upper and lower blood/glass interfaces determined when the channel is filled with blood with the width of the flow channel. The value obtained for the optical index of refraction for blood at 1300 nm by this method is *n*_blood_ = 1.39 ± 0.05. The uncertainty in the measured optical refractive index arises from both the specified tolerance in the width of the flow cell channel, 0.01 mm (Starna Inc. catalog), and the imprecision introduced by the finite coherence length of the OCT source, ~0.014 mm.

Another two-dimensional OCT image, this time of blood flowing through the 2-mm flow cell, is presented in Figure 1(b). As light penetrates deeper inside blood, it is reflected, scattered, and absorbed by various cellular aggregates and fluidic blood components. Due to the strong light scattering environment that is flowing blood, there is no recorded OCT signal that comes from the second glass/interface, that is, the interface located at a 2-mm distance from the interface through which light enters blood. It is known that while light propagates through blood, it undergoes diffuse reflection from the blood cell membranes and therefore the backscattered OCT signal is weak. Also, according to [11, 20], the scattering anisotropy factor for blood measured at various wavelengths is reported to be between 0.94 and 0.995. Such values of the anisotropy factor are characteristic to a strong forward scattering of light in blood and translate into a reduced probability for its backscattering toward the OCT detection system. The cumulative effect of multiple scattering events is another factor reducing the detected signal. By undergoing multiple interactions within the turbid environment, part of the probing light flux is pushed out from the field of view of the objective lens or beyond the spatial detection gate imposed by the optical delay line in the reference arm. These factors, plus the low power of the interrogating beam, contribute to an OCT signal that is detected after probing a maximum distance of only 0.25 mm within blood.

**Figure 2:**
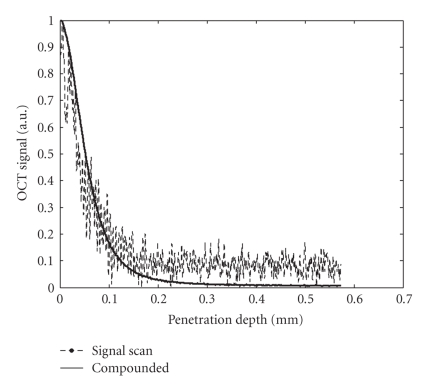
Single OCT depth-line scan (dotted line) compared to the compounded profile (continuous curve). The single-scan line is the 600th A-scan marked with a white interrupted line in Figure 1(b). The compounded profile results from the summation of 1000 consecutive A-scans.

An example of a demodulated A-scan interferogram is indicated in Figure 2 by the dotted line. It shows the signal generated into the OCT detection system through backscattering of light by blood cells during the 600th A-scan. The position of this particular A-scan is marked with a white interrupted line in the two-dimensional OCT image from Figure 1(b). In Figure 2, the specular reflection peak corresponding to the glass/blood interface is eliminated in order to better emphasize the signal generated only by the diffuse reflections occurring within the blood environment. The noisy profile of the single A-scan is common for OCT scans of highly scattering media and can be attributed to the random positions of scattering centers and to the speckle noise generated by multiple scattering. Speckle noise occurs in OCT imaging applied to turbid media because of multiple scattered light that experiences changes in the travel distance relative to its initial ballistic path reaching the detection system [17, 21]. In order to accurately estimate the propagation properties of light in a highly scattering medium, it is necessary to reduce the speckle noise. In OCT images, speckle noise can be suppressed by adding spatially independent scans [22–24]. In the presented case, because of the blood flow, there is a dynamic distribution of cellular aggregates that act as scattering centers, which in turn induces variations of the speckle pattern from one A-scan to another. Adding (compounding) independent individual A-scans with uncorrelated speckle patterns results into a smoothed depth profile where some of the speckle noise as well as the noise generated by random electronic and thermal variations in the OCT detection system are eliminated. Such a compounded profile is exemplified by the continuous curve from Figure 2, which is obtained by compounding 1000 individual A-scans. To account for the scaling difference between profiles, the single scan as well as the compounded one is normalized to unity in Figure 2.

As light penetrates deeper inside blood, the recorded OCT signal decreases due to both optical absorption and scattering. Within the depths probed in our experiment, scattering is predominantly responsible for dissipation of the near-infrared signal while absorption is responsible for less than 0.5% of the total radiation loss [17]. Therefore, in the following we will consider that the profiles of individual and compounded depth scans are exclusively shaped by the scattering properties of blood.

The aggregate recorded OCT signal power, 〈*i* ^2^(*z*)〉, corresponding to the OCT signal received from a given depth, *z* , inside the blood environment can be expressed as the product between the mean square heterodyne signal in the absence of scattering, 〈*i* ^2^〉_0_, and the heterodyne efficiency, presented as the sum of three terms that account for the scattering losses [25]:

(1) $$\langle i^{2}\left(z\right)\rangle \;=\;{\langle i^{2}\rangle }_{0}\times \;[\exp\left(-2\mu z\right)\;+\;\frac{4\exp\left(-\mu z\right)\left[1\;-\;\exp\left(-\mu z\right)\right]}{1\;+\;r^{2}\left(z\right)}\;+\;\frac{{\left[1\;-\;\exp\left(-\mu z\right)\right]}^{2}}{r^{2}\left(z\right)}].$$

In (1), *μ* represents the scattering coefficient and *r* ^2^ is the halo parameter, that is, the ratio between the squares of the *1/e* irradiance radii in the *z*-plane in the presence and absence of scattering. The halo coefficient is a measure of the lateral coherence length including its dependence on the penetration distance, that is, the shower curtain effect [25].

As shown by the compounded profile in Figure 2, the light penetration in blood for which a readable OCT signal is obtained for this particular experimental configuration is approximately 0.25 mm, which is small when compared to the focal distance (48 mm) of the objective lens used in the sample arm. Besides the single-backscattered component, there is also multiple-scattered signal that is recorded in our OCT measurements despite the low depth of penetration into the turbid environment [26–28]. The following fact has to be considered in our analysis: blood cells are not point-like scattering centers but disk-like entities with diameters around 8 *μ*m and thicknesses of approximately 3 *μ*m that occupy a certain volume in space to the extent that light spends an amount of time propagating through the cell ensemble comparable to the time spent propagating into the blood plasma. Therefore, because of intra- and intercell multiple light reflections occurring at the cell/plasma interfaces, OCT signal that experienced multiple scattering events is recorded even after short distances (tens of *μ*m) of propagation through blood. The early onset of a multiple-scattered component in the recorded signal generated when light propagates in blood phantoms or blood/saline mixtures has been documented in previous publications [26–28]. Also, the amount of multiple scattered signal increases as the OCT signal is recorded from deeper within the scattering environment [26–28].

In our experimental configuration, the depth within blood that is probed is centered at the waist of the weakly focused probe beam. Therefore, the lens-induced axial variation in its intensity has a minimal impact on the recorded OCT profile. Avoiding the influence of the beam divergence on the intensity of the compounded profile is helped also by the small value of the numerical aperture of the optical system from the OCT sample arm, that is 0.025.

Within these experimental conditions and taking into account the configuration of the OCT system sample arm, the halo factor can be expressed as

(2) $$r^{2}\left(z\right)\;=\;1\;+\;C\mu z^{3}.$$

In (2), *C* is a constant depending on sample parameters like the blood refraction index and the scattering anisotropy parameter, *g,* as well as on instrumental constants: the effective numerical aperture, N.A., of the objective lens used to focus the probing beam onto the sample and the free-space wavelength of the source, *λ*:

(3) $$C\;=\;\frac{8\pi^{2}}{3}{\left(\frac{\text{N}.\text{A}.}{\lambda }\right)}^{2}\frac{\left(1\;-\;g\right)}{n_{\text{blood}}^{2}}.$$

**Figure 3:**
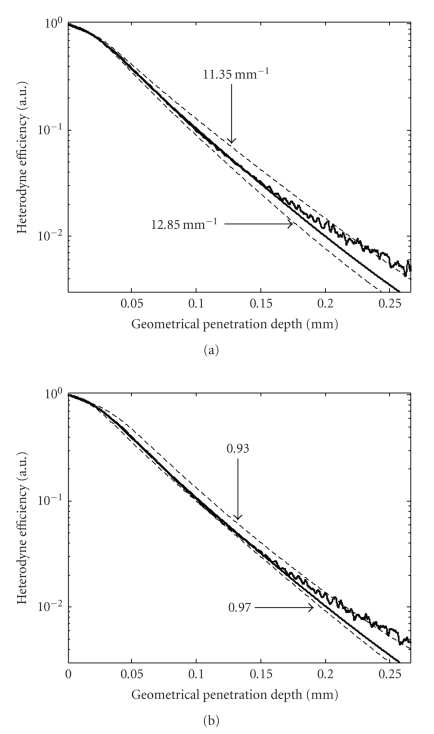
Best numerical fit with the experimental heterodyne profile is obtained for values of *μ* = 12.15 mm^−1^ and *g* = 0.95 for the attenuation coefficient and anisotropy parameter, respectively. The numerical fit is the middle line that follows closely the experimental heterodyne efficiency curve shown in both (a) and (b) parts of the figure. (a) Variations of the numerical fit induced by changes of ±0.7 mm^−1^ in the attenuation coefficient with the anisotropy scattering factor kept at *g* = 0.95. (b) Variations in the numerical fit induced by variations of ±0.02 in the scattering anisotropy parameter with the attenuation coefficient kept at *μ* = 12.15 mm^−1^.

The term contained in the brackets in relation (1), that is, the heterodyne efficiency which can be also expressed as the 〈*i* ^2^(*z*)〉/〈*i* ^2^〉_0_ ratio, is fit with the experimentally measured and normalized compounded OCT signal power shown in Figures 3(a) and 3(b). Having previously measured the index of refraction of blood and knowing the wavelength as well as the effective numerical aperture, the latter precisely measured using the technique described in [29, 30], only the scattering coefficient and the anisotropy parameter are used as fitting parameters. The numerical fit with the experimental heterodyne efficiency curve is performed up to the point where the signal is 1.5 times higher than the detection shot noise. Values of 12.15 mm^−1^ and 0.95 are obtained for the total attenuation coefficient and for the scattering anisotropy factor. These values are consistent with the values obtained in [11]. When compared to results determined at visible and shorter near-infrared wavelengths, the trend of decreasing scattering coefficients for longer wavelengths in the near-infrared region is also confirmed. This trend has been both experimentally observed and theoretically predicted in several publications [11, 20, 31].

The anisotropy factor estimated in this work by applying the extended Huygens-Fresnel principle to the compounded OCT profile has a value that is approximately 4% smaller than values theoretically calculated or measured from transmission-type experimental arrangements. In such experimental setups, the amount of scattered flux is measured or calculated at detection points located at distances much greater than the dimensions of the volume where the scattering takes place. Therefore, the signatures of individual scattering centers, blood cells in the case under study, are lost because only the far-field flux is measured. The far-field flux is a quantity that is an averaged result of the overall scattering events occurring in the entire probed volume. Meanwhile, due to the interference-based axial sectioning capabilities and due to the coherence-imposed limitations on the detected signal, only the scattering properties of blood constituents from specific locations confined within a small volume are assessed with an OCT-based configuration. More clearly, in an OCT configuration the detected signal can be envisioned as portioned into units that are the result of light interacting only with a small number of cells located within a sample volume defined by the coherence length of the OCT source and the area of the probing beam. Therefore, the detected OCT signal is sensitive to the optical and geometrical characteristics of the individual blood cell and the morphological discontinuities occurring on microscopic scales can affect its outcome.

Figures 3(a) and 3(b) help in determining the sensitivity of the fitting procedure to small variations of the attenuation coefficient and anisotropy scattering parameter. For comparison, curves corresponding to attenuation coefficients of 11.35 mm^−1^ and 12.85 mm^−1^ are calculated and shown in Figure 3(a) with the anisotropy parameter kept at 0.95, the value obtained from the numerical fit. Similarly, curves with two different anisotropy scattering factors, 0.97 and 0.93, are plotted in Figure 3(b) using the attenuation coefficient numerically obtained from fitting the compounded OCT profile. Differences between the curves obtained with these values of the attenuation coefficient and anisotropy factor and the curve that provides the best numerical fit to the experimental data are obvious from the figure. The values used to derive the unfit curves are used to estimate the precision of the procedure. The root-mean-squared error (RMSE) provides a quantitative measurement of the deviation of the numerical models from the experimental data. The smallest RMSE is of course obtained for the numerical fit. As a comparison among the other four cases, the smallest RMSE is obtained for *g* = 0.97. That RMSE value is still 35% greater than the one corresponding to the numerical fit.

## 4. CONCLUSIONS

A method based on the extended Huygens-Fresnel principle applied to time-domain OCT measurements is demonstrated to directly estimate optical parameters of blood. By employing this procedure, the use of phantom samples prepared under predetermined physiological conditions as well as complicated reconstruction algorithms could be avoided. The research presented here fills a knowledge gap regarding the optical interaction between blood constituents and light at 1300 nm. Knowing the refractive index of blood and the attenuation induced in the signal by light scattering enables a more precise estimation of the effective propagation distance of OCT signal into blood for a system configuration similar to an OCT-based intravascular catheter design. The effective depth that can be probed within blood before the signal becomes imbedded into the shot noise is a useful parameter to be known when designing and using OCT-based intravascular catheters. Values of 12.15 mm^−1^, 1.39, and 0.95 are estimated for the total attenuation, the refraction index, and the scattering anisotropy factor, respectively. Both the attenuation coefficient and the anisotropy factor are determined simultaneously by applying the extended Huygens-Fresnel principle to the experimental heterodyne efficiency curve. The refraction index of blood is determined to a second decimal precision while the total attenuation coefficient and the scattering anisotropy parameter values are within experimental error margins of 5% and 2%, respectively.
